# Trimodality strategy for treating malignant pleural mesothelioma: results of a feasibility study of induction pemetrexed plus cisplatin followed by extrapleural pneumonectomy and postoperative hemithoracic radiation (Japan Mesothelioma Interest Group 0601 Trial)

**DOI:** 10.1007/s10147-015-0925-1

**Published:** 2015-11-17

**Authors:** Seiki Hasegawa, Morihito Okada, Fumihiro Tanaka, Takeharu Yamanaka, Toshinori Soejima, Norihiko Kamikonya, Tohru Tsujimura, Kazuya Fukuoka, Kohei Yokoi, Takashi Nakano

**Affiliations:** Department of Thoracic Surgery, Hyogo College of Medicine, 1-1 Mukogawa-cho, Nishinomiya, 663-8501 Japan; Department of Surgical Oncology, Hiroshima University, Hiroshima, Japan; Department of Surgery, University of Occupational and Environmental Health, Kitakyusyu, Japan; Department of Biostatistics, Yokohama City University, Yokohama, Japan; Department of Radiation Oncology, Hyogo Cancer Center, Akashi, Japan; Department of Radiation Oncology, Hyogo College of Medicine, 1-1 Mukogawa-cho, Nishinomiya, 663-8501 Japan; Department of Pathology, Hyogo College of Medicine, 1-1 Mukogawa-cho, Nishinomiya, 663-8501 Japan; Department of Medical Oncology, Sakai Hospital, Kinki University Faculty of Medicine, Osaka, Japan; Department of Thoracic Surgery, Nagoya University Graduate School of Medicine, Nagoya, Japan; Division of Respiratory Medicine, Department of Internal Medicine, Hyogo College of Medicine, 1-1 Mukogawa-cho, Nishinomiya, 663-8501 Japan

**Keywords:** Mesothelioma, Clinical trials, Pleural disease, Thoracic surgery

## Abstract

**Purpose:**

We conducted a prospective multi-institutional study to determine the feasibility of trimodality therapy (TMT) comprising induction chemotherapy followed by extrapleural pneumonectomy (EPP) and radiation therapy in Japanese patients with malignant pleural mesothelioma (MPM).

**Methods:**

Major eligibility criteria were histologically confirmed diagnosis of MPM, including clinical subtypes T0–3, N0–2, M0 disease; no prior treatment for the disease; age 20–75 years; Eastern Cooperative Oncology Group performance status 0 or 1; predicted postoperative forced expiratory volume >1000 ml in 1 s; written informed consent. Treatment methods comprised induction chemotherapy using pemetrexed (500 mg/m^2^) plus cisplatin (60 mg/m^2^) for three cycles, followed by EPP and postoperative hemithoracic radiation therapy (54 Gy). Primary endpoints were macroscopic complete resection (MCR) rate for EPP and treatment-related mortality for TMT.

**Results:**

Forty-two eligible patients were enrolled: median age 64.5 (range 43–74) years; M:F = 39:3, clinical stage I:II:III = 14:13:15; histological type epithelioid were sarcomatoid; biphasic; others = 28:1:9:4. Of 42 patients, 30 completed EPP with MCR and 17 completed TMT. The trial met the primary endpoints, with an MCR rate of 71 % (30/42) and treatment-related mortality of 9.5 % (4/42). Overall median survival time and 2-year survival rate for 42 registered patients were 19.9 months and 42.9 %, respectively. Two-year relapse-free survival rate of 30 patients who completed EPP with MCR was 37.0 %.

**Conclusion:**

This phase II study met the predefined primary endpoints, but its risk/benefit ratio was not satisfactory.

**Electronic supplementary material:**

The online version of this article (doi:10.1007/s10147-015-0925-1) contains supplementary material, which is available to authorized users.

## Introduction

Malignant pleural mesothelioma (MPM) is a rare and locally aggressive tumor with a median survival time (MST) of 9–12 months [1]. MPM is associated with asbestos exposure, and its incidence is highly correlated with asbestos exposure, with a latency of 30–40 years [2]. The incidence of this disease peaked around 2004 in the United States and will peak from 2015 to 2020 in Europe and Australia [3]. However, in Japan, the peak may occur from 2030 to 2035 because of a historical delay in the heavy use of asbestos [4, 5]. High asbestos exposure in developing countries, particularly in Asia, is likely to cause future disease; however, this is difficult to quantify [6, 7].

Treating MPM is challenging. Extrapleural pneumonectomy (EPP) is performed with curative intent, but the outcome is not acceptable in patients treated with surgery alone. Accordingly, the curative strategy in patients with resectable MPM shifted to trimodality therapy (TMT), which comprises induction chemotherapy followed by EPP and radiation therapy (RT). Evidence from studies conducted in North America and Europe on TMT suggests that this strategy is feasible but has a poor risk-to-benefit ratio. Here we report the results of the Japan Mesothelioma Interest Group (JMIG) 0601 Trial, a prospective multi-institutional study to evaluate the feasibility of TMT for Japanese patients with MPM.

## Patients and methods

### Patients

Patients were eligible [8] if they had a histologically confirmed diagnosis of MPM that was considered resectable, including all subtypes and clinical stage T0-3, N0-2, M0 disease, according to the International Mesothelioma Interest Group (IMIG) Staging System [9]. Other requirements were as follows: age between 20 and 75 years; an Eastern Cooperative Oncology Group (ECOG) performance status of 0–1; adequate bone marrow, hepatic, renal, cardiac, and respiratory functions; a predicted postoperative forced expiratory volume of ≥1000 ml in 1 s; and written informed consent. Existence of a measurable lesion was not mandatory. Exclusion criteria included any prior treatment for MPM, and serious systemic complications, including poorly controlled diabetes or hypertension, active infectious disease, interstitial pneumonia or lung fibrosis, simultaneous or two metachronous (within 5 years) cancers, serious drug allergy or hypersensitivity to any drugs, pregnancy or breastfeeding, and grade 2 or greater peripheral neuropathy at registration. The ethics committee of each participating center approved the protocol before patients were enrolled.

### Endpoints

The primary endpoints comprised macroscopic complete resection (MCR) rate by EPP and treatment-related mortality. MCR was defined as surgical removal of all gross tumor tissue [10]. Treatment-related death (TRD) was defined as any death occurring within 84 days after cessation or completion of TMT not related to disease progression. Secondary endpoints were proportion of patients who completed TMT, incidence of adverse events (AEs) during TMT, response rate for induction chemotherapy, 2-year overall survival rate of all eligible patients, and 2-year relapse-free survival (RFS) of patients with MCR.

### Therapeutic regimens

The treatment protocol of this study comprised induction chemotherapy followed by surgery and adjuvant radiotherapy.

### Chemotherapy

Induction chemotherapy consisted of three cycles of pemetrexed (500 mg/m^2^) followed by cisplatin (60 mg/m^2^) on day 1 and was given every 21 days. Folic acid (0.5 mg per day, orally) and vitamin B_12_ (1 mg intramuscularly every 9 weeks) were administered 1–3 weeks before the first dose of chemotherapy and continued throughout induction chemotherapy. Dose adjustments were required for renal and nonhematologic toxicity as well as hematologic effects. A dose delay up to 42 days was permitted for recovery from drug toxicity. Tumor response was assessed using computed tomography (CT) after completion of induction chemotherapy using a modified version of the Response Evaluation Criteria in Solid Tumors (mRECIST) [11].

### Surgery

EPP was performed within 42 days following the last dose of induction chemotherapy unless there was progression of disease or deterioration of organ functions that would make the surgery intolerable. EPP was defined as an en bloc resection of the entire pleura and the ipsilateral lung, with resection of the ipsilateral diaphragm, pericardium, or both, if required [12]. A systematic hilar and mediastinal lymphadenectomy was preferred. Protocol treatment was terminated for patients with unresectable tumor at thoracotomy.

### Radiotherapy

Adjuvant hemithoracic RT was performed within 12 weeks after surgery according to the methods described by Rusch et al. [13]. Patients received three-dimensional conformal RT using a linear accelerator that delivered 6–20 MV photon and electron beams. A total dose of 54 Gy in 30 fractions of 1.8 Gy/day was delivered. The clinical target volume (CTV) included the entire ipsilateral hemithorax pleural space and chest-wall incisions. If regional lymph nodes were involved, CTV included mediastinal lymph nodes. The planning target volume (PTV) included the CTV and a margin around the CTV to compensate for errors in treatment setup and internal target motion during treatment. Cerrobend blocks limited the dose to the liver, heart, and stomach, and electrons were used in blocked regions to dose the diaphragm and chest wall adequately. The spinal cord was protected for doses >41.4 Gy. The dose–volume planning objectives defined for the organs at risk were as follows: contralateral lung, percentage of volume receiving 5 Gy (V5) <50 %, V20 <7 %; liver, V20 <50 %; heart, V45 <50 %; kidney, V15 <20 %; spinal cord, maximum dose <48 Gy. Intensity-modulated RT was not allowed in this study.

### Evaluation

When TMT was completed or when protocol treatment ceased, patients underwent a physical examination and chest radiography/CT scans every 3 months up to 3 years or until death. If recurrence of MPM was suspected, appropriate examinations were performed. Major complications caused by chemotherapy, surgery, or RT were scored according to the National Cancer Institute Common Terminology Criteria for Adverse Events version 4.0 guidelines [14].

### Study design and statistical methods

Sample size was determined according to the results of an exact binominal test for MCR rate (%MCR). The target size of 40 eligible patients assured a statistical power of approximately 0.90 when expected and acceptable lowest %MCRs were 70 and 50, respectively, with a one-sided level of 0.10. Treatment-related mortality of 10 % was considered as the acceptable upper limit. Termination of the study was planned if the total number of TRDs reached five, indicating that a point estimate for treatment-related mortality exceeded 10 % of the planned sample size. Interim analyses were performed according to the Bayesian predictive probability for %MCR. SAS version 9.2 (SAS Institute Inc, Cary, NC, USA) and GraphPad Prism version 6.0 for Windows (GraphPad Software, San Diego, CA, USA) were used for analyses. A *P* value of <0.05 was considered statistically significant.

### Study monitoring

The Data and Safety Monitoring Committee independently monitored protocol compliance and study progress and reviewed interim analysis reports. The study was registered at the University Hospital Medical Information Network (UMIN) Clinical Trials Registry (No. 000001154) in May 2008.

## Results

### Baseline characteristics

**Fig. 1:**
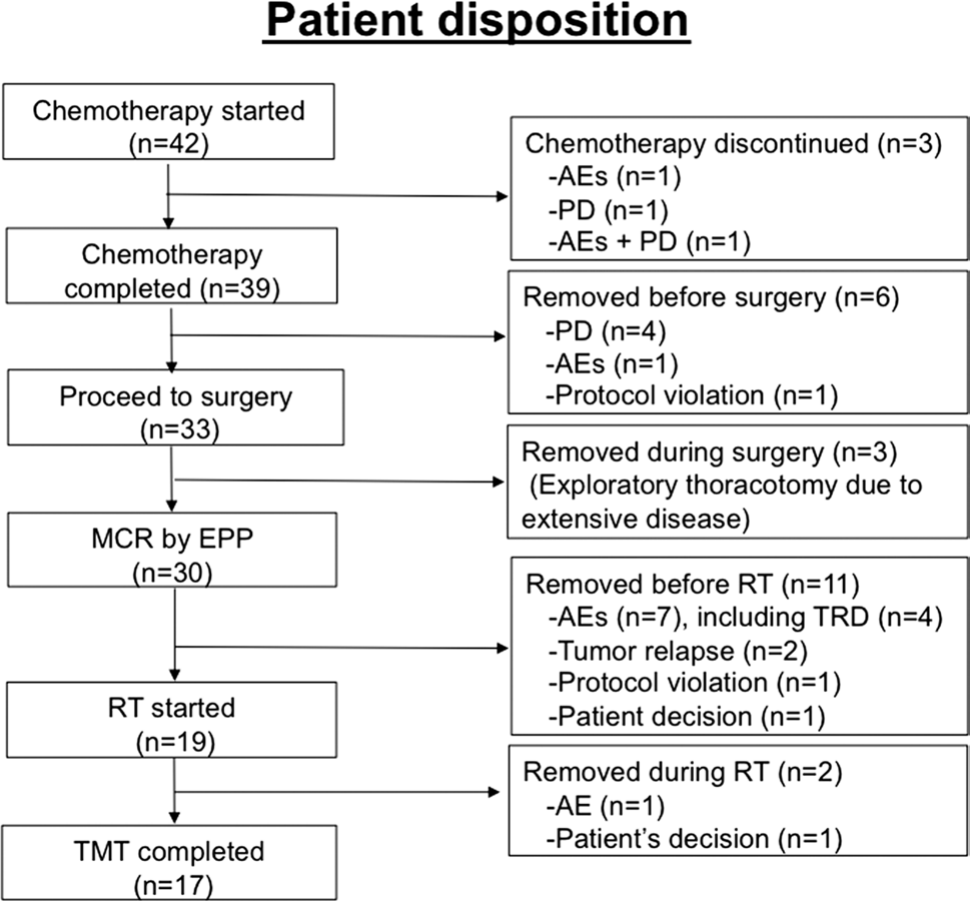
Consolidated Standards of Reporting Trials (CONSORT) diagram of registered patients. Of 42 registered patients, 33 underwent surgery. Three patients underwent exploratory thoracotomy because of unexpected extensive disease, and the remaining 30 completed extrapleural pneumonectomy (EPP) achieved macroscopic complete resection (MCR). Nineteen patients who underwent EPP started radiotherapy (RT), which was completed by 17

Forty-two patients were enrolled from 12 institutions in Japan between May 2008 and November 2010. All patients were eligible. Patients’ characteristics are shown in Table 1. The Consolidated Standards of Reporting Trials (CONSORT) diagram is summarized in Fig. 1.

**Table 1:** Patient characteristics

Characteristics	Number of patients	Percentage
Age		
Median	65	
Range	43–75	
Sex		
Male	39	92.9
Female	3	7.1
Location		
Right	19	45.2
Left	23	54.8
Histology		
Epithelial	28	66.7
Sarcomatoid	1	2.4
Biphasic	9	21.1
Indeterminate	4	9.5
Clinical stage		
T1N0M0	15	
T2N0M0	12	
T2N1M0	2	
T2N2M0	2	
T3N0M0	7	
T3N1M0	1	
T3N2M0	3	
Pathological stage^a^		
T1N0M0	4	
T2N0M0	7	
T3N0M0	12	
T3N1M0	1	
T3N2M0	5	
T4N0M0	1	

^a^Patients who completed extrapleural pneumonectomy (*n* = 30)

### Chemotherapy

Neoadjuvant chemotherapy was started in 42 patients and discontinued in three because of progressive disease (*n* = 1), AE (*n* = 1), or both (*n* = 1). The remaining 39 patients completed three courses of induction chemotherapy without requiring any dose reduction. Thus, the chemotherapy completion rate was 92.9 % (39/42). There was one case with grade 4 toxicity of neutropenia (Table 2). Grade 3 toxicity was observed in seven patients. There were no chemotherapy-induced deaths. According to the mRECIST criteria, 14 patients achieved partial remission (PR), 19 had stable disease (SD), and seven had progressive disease (PD). Two patients were not evaluable because they had no radiologically evaluable lesions. Response and disease-control rates were 33.3 % [95 % confidence interval (CI) 19.6–49.6 %] and 78.6 % (95 % CI 63.2–89.7 %), respectively.

**Table 2:** Chemotherapy-related adverse events

Adverse event	Grade 1	Grade 2	Grade 3	Grade 4
Hematologic				
Neutropenia	8	4	1	1
Thrombocytopenia	2			
Nonhematologic				
Nausea/appetite loss	23	11	2	
Vomiting	4	2	1	
Fatigue	8	3		
Diarrhea	2			
Constipation	9			
Febrile neutropenia	1			
Dyspnea	3			
Neurologic disorder	2	1		
Hiccup	6	1		
Radiculopathy		1		
Liver dysfunction	1		1	
Hyperglycemia			2	

### Surgery

Of the 39 patients who completed chemotherapy, six did not proceed to EPP because of progressive disease (*n* = 4), AE (*n* = 1), or protocol violation (*n* = 1). EPP was started in 33 patients and was completed in 30 (14 right sided, 16 left sided). These 30 patients achieved MCR with reconstruction of the diaphragm (*n* = 2), pericardium (*n* = 2), or both (*n* = 26). EPP was abandoned in three patients because of extensive chest wall invasion (*n* = 2) or transmural pericardial invasion (*n* = 1). Accordingly, the MCR rate was 71.4 % (30/42). Median operation time and blood loss in completed EPP cases (*n* = 30) were 437 (range 335–655) min and 1461 (range 390–4530) g, respectively.

There were four TRDs, and all occurred within 84 days after EPP (Table 3). One patient died of bronchopleural fistula, empyema, and acute respiratory disease syndrome (ARDS) on postoperative day (POD) 30. Another patient developed cardiac herniation and hemothorax on POD 23 and died on POD 80. Two patients died of ARDS on PODs 61 and 72.

**Table 3:** Treatment-related deaths (*n* = 4)

Patients	1	2	3	4
Age	66	72	69	72
Sex	Male	Male	Male	Male
Side	Right	Right	Right	Left
Histology	Epithelioid	Epithelioid	Intermediate	Biphasic
Clinical stage	II	I	III	III
Asbestos exposure	None	Yes	Yes	Yes
Smoking (pack-year)	34	0	0	50
Preoperative risks	None	Atrial fibrillation	None	None
Response to chemotherapy	PR	SD	PR	SD
Operation time (min)	362	439	366	410
Operative blood loss (g)	760	3950	390	4360
AE	ARDS	ARDS	BPF, ARDS	Cardiac herniation
AE onset (days after EPP)	41	28	4	23
Death (days after EPP)	61	72	30	80

*AE* adverse event,* EPP* extrapleural pneumonectomy,* PR* partial response,* SD* stable disease,* ARDS* acute respiratory distress syndrome

Postoperative complications are summarized in Table 4. Grade 3 or greater postoperative complications occurred in 21 of 33 patients (63.6 %). Arrhythmia occurred in 11 (33.3 %) but was not critical in all cases. Bronchopleural fistula developed in three patients who underwent right-sided EPP.

**Table 4:** Surgery-related adverse events

Adverse event	Grade 1	Grade 2	Grade 3	Grade 4
Bronchopleural fistula				3
Empyema			4	
Chylothorax			1	
Diaphragmatic patch dislocation			3	
Pneumonia		1	2	
ARDS/interstitial pneumonia		1		2
Dyspnea	4	2	1	2
Bleeding/hemothorax		1	2	1
Heart failure		1	3	1
Shock			2	1
Arrythmia	3	8		
Delirium				1
Fatigue			2	
Hoarseness	1			

*ARDS* acute respiratory distress syndrome

### Radiation

Of the 30 patients with MCR, 11 did not proceed to RT because of AEs (*n* = 7), tumor relapse (*n* = 2), protocol violation (*n* = 1), or personal decision (*n* = 1). Of the 19 patients who started RT, two discontinued because of AE (*n* = 1) or refusal (*n* = 1). Accordingly, TMT was completed in 17 (40.5 %, *n* = 42) patients. Grade 3 toxicity related to RT occurred in four patients (Table 5). There were no patients with grade ≥4 AEs or grade ≥3 radiation pneumonitis or interstitial pneumonia.

**Table 5:** Radiation-related adverse events

Adverse event	Grade 1	Grade 2	Grade 3	Grade 4
Nausea/appetite loss	6	4	3	
Vomiting	1	2		
Pneumonia			1	
Esophagitis	5	6		
Dermatitis	7	3	1	
Fatigue	7	4	2	

### Histology

Preoperative histological diagnosis of epithelioid MPM was revised to biphasic MPM postoperatively for two out of 30 patients who completed EPP. In the other three patients with a preoperative diagnosis of undetermined MPM, two were diagnosed as having sarcomatoid and one as having epithelioid MPM.

### Survival, RFS, and recurrence

**Fig. 2:**
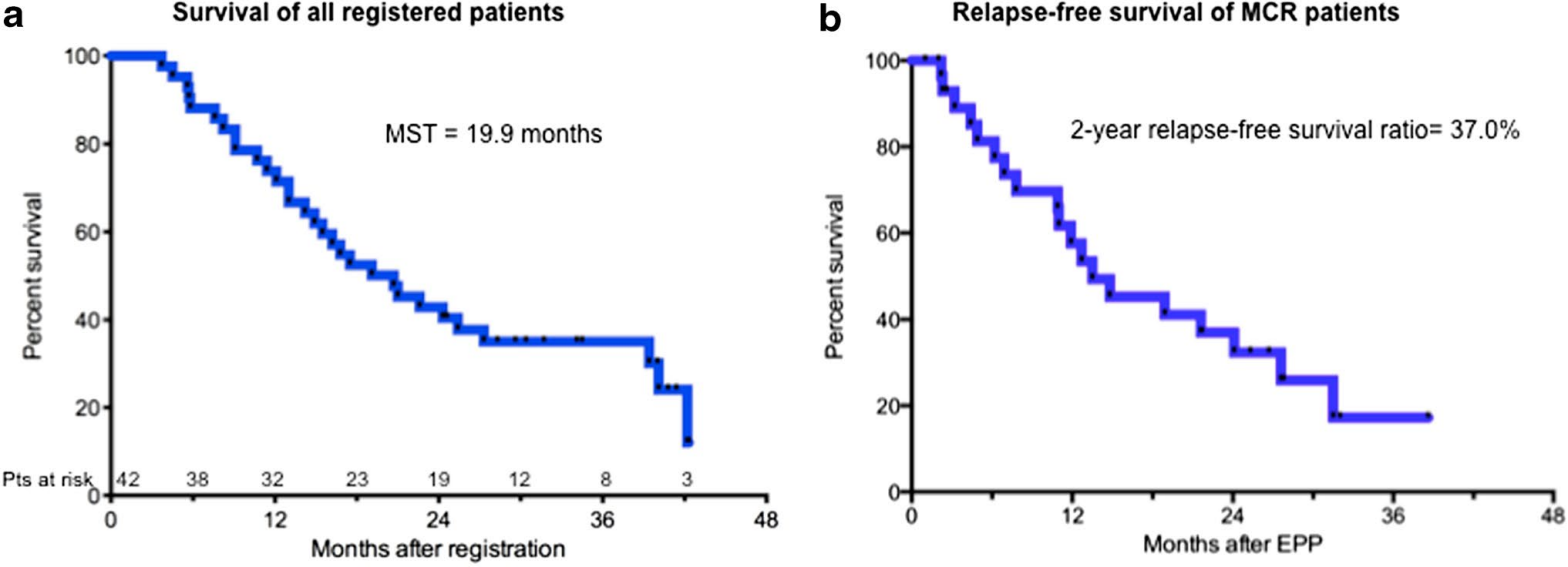
Survival. **a** Kaplan–Meier analysis of the overall survival of 42 intent-to-treat (ITT) patients [median 19.9 months; 95 % confidence interval (CI) 14.2–27.3 months]. **b** Relapse-free survival (RFS) of extrapleural pneumonectomy (EPP) patients. Median RFS of patients who completed EPP with macroscopic complete resection (MCR) was 11.0 months (95 % CI 2.2 %–31.5 % months). RFS rate 2 years after surgery was 57.6 %

**Fig. 3:**
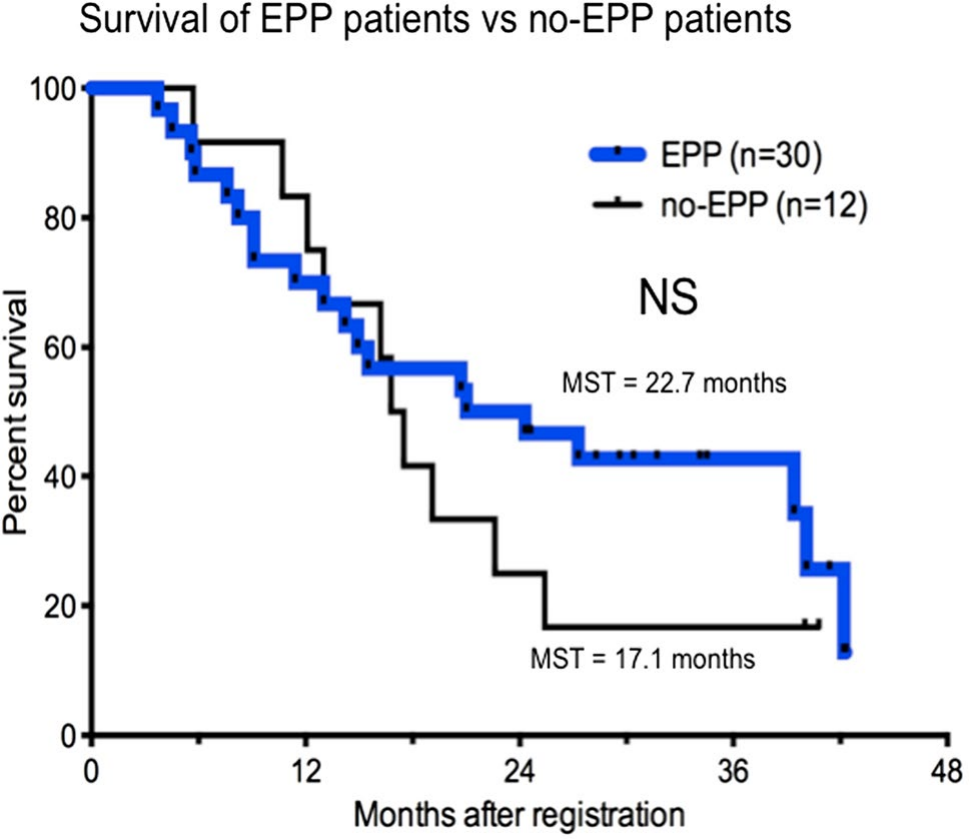
Comparison of Kaplan–Meier overall survival analyses of extrapleural pneumonectomy (EPP) versus non-EPP patients. No significant difference was observed (median 22.7 months for EPP patients vs. 17.1 months for non-EPP patients)

The median follow-up period was 19.9 months after registration. MST and the 2-year survival rate for all registered patients (*n* = 42) were 19.9 (95 % CI 14.2–27.3 %) and 42.9 % (95 % CI 27.8–57.1 %) months, respectively (Fig. 2a). MST and the 2-year survival rate of patients who completed EPP (*n* = 30) were 22.7 months and 50.0 %, respectively, and those for non-EPP patients (*n* = 12) were 17.1 months and 25.0 %, respectively (Fig. 3).

Among the 30 patients who completed EPP with MCR, 19 relapsed postoperatively, with a median RFS of 11.0 (95 % CI 2.2–31.5 %) months; RFS rates 1 and 2 years after surgery were 57.6 and 37.0 %, respectively (Fig. 2b). Relapse occurred at ipsilateral pleural effusion/chest wall (*n* = 8), mediastinum/pericardial effusion (*n* = 7), contralateral lung parenchyma (*n* = 2), ascites (*n* = 3), lymph nodes (*n* = 3), and unknown (*n* = 1). Of 17 patients who completed TMT, relapse occurred in 11 at ipsilateral pleural effusion/chest wall (*n* = 5), mediastinum/pericardial effusion (*n* = 2), contralateral lung parenchyma (*n* = 2), and ascites (*n* = 2). Significantly longer survival was observed in patients with a preoperative diagnosis of epithelioid histology compared with nonepithelioid histology (MST 27.3 vs. 13.6 months, *P* = 0.0013), and there were no statistically significant differences in survival according to other preoperative variables, such as age, gender, side, histology, clinical stage, and radiological response to chemotherapy.

**Fig. 4:**
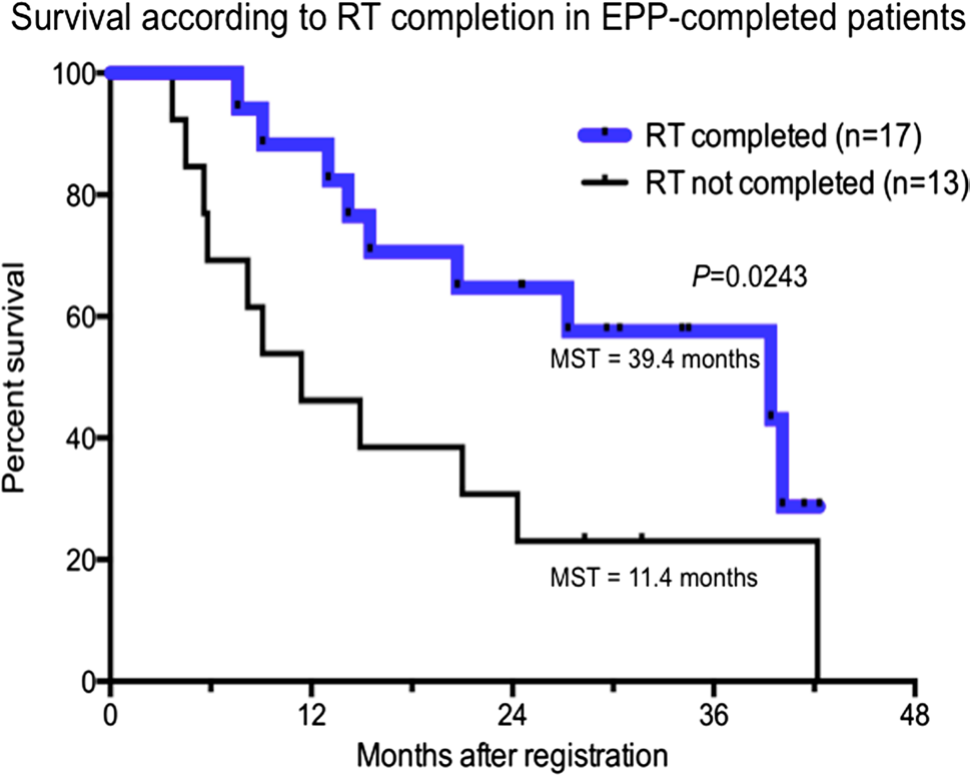
Significantly longer survival was observed for patients who completed trimodality therapy (TMT) (*n* = 17) in comparison with patients who completed extrapleural pneumonectomy (EPP) but not TMT (*n* = 13)

There was no statistically significant survival difference correlated with pathological T/N factors and pathological stage. Significantly longer survival was observed for patients who completed TMT (*n* = 17) in comparison with patients who completed EPP but not TMT (*n* = 13) (MST 39.4 vs. 11.4 months, *P* = 0.0243) (Fig. 4).

## Discussion

To date, several prospective studies have been conducted in North America and Europe in which MPM was treated by neoadjuvant chemotherapy followed by EPP and hemithoracic RT [15–21]. Treatment regimen in the study reported here was almost equivalent to that employed by the North American [19] and European [20] studies, with exception of cisplatin dose (Table 6, online only). Cisplatin dose of 60 mg/m^2^ used here was based on results of studies conducted in Japan for MPM treatment [22, 23]. Because efficacy and safety of the combination of cisplatin and other drugs were assessed and confirmed in those studies, we administered cisplatin intravenously (60 mg/m^2^) in combination with pemetrexed.

The aims of our study were to determine whether TMT for MPM was feasible in Japan. Although all 12 participating institutions are leading treatment centers in Japan, none had experienced >20 cases of EPP during the 5 years prior to entering this study. Because the two primary endpoints were met here, we verified that TMT for MPM is feasible in Japan. Completion rates of induction chemotherapy, EPP, and MCR were comparable with those of the US trial [19] and EORTC 08031 [20]. However, there are striking differences in the number of patients who failed to proceed to RT after completion of EPP, e.g., 26 % of the intent-to-treat (ITT) population in the study presented here versus 13 and 7 % in the US trial and EORTC 08031, respectively. This may be explained by the less stable condition of our patients after surgery, reflecting less experience with TMT of the participating Japanese centers. Racial differences in resistance to highly aggressive treatment between Japanese and Caucasians may explain the different outcomes as well [24]. However, this argument is hardly convincing, because mortality due to pneumonectomy in patients with lung cancer in Japan [25] is approximately one third of that in the US [26] or UK [27].

Another aim of the study was to determine whether survival after TMT of Japanese patients with MPM was comparable with that of Caucasian patients. MST and RFS in this study were comparable with those of previous studies, notwithstanding the lower rate of TMT completion. This may partly indicate that MST of patients who completed TMT was longer in this study (39.4 months) compared with that of patients in US (29.1 months) [19] and European (33.0 months) [20] studies. However, the role of RT remained unclear, since the relapse pattern was similar in patients with or without RT in this study.

The study reported here verifies that TMT in patients with MPM is feasible in Japan, with similar survival and risk rates compared with those conducted in North America and Europe. However, it should be emphasized that the risk-to-benefit ratio in our study, as well as in the US/European studies, is not satisfactory.

Several centers tend to choose pleurectomy/decortication (P/D) instead of EPP as curative-intent surgery [28]. This trend appears to be related to the following situation: EPP is disadvantageous because of its high risk and occurrence of postoperative cardiopulmonary deterioration [29, 30], and it only provides comparable postoperative survival with P/D [31, 32]. Note that the narrative above is based on retrospective nonrandomized studies because there is no prospective randomized study that directly compares EPP and P/D. Furthermore, there are very few completed prospective phase II studies of P/D [33, 34].

In this context, the JMIG 1101 Trial, a multi-institutional, single-arm, feasibility study of induction pemetrexed plus cisplatin followed by P/D was conducted in Japan [35], and patient recruitment was completed by January 2014. Although a direct comparison of results of JMIG 1101 and our study is not appropriate, it may provide valuable information regarding the choice between EPP and P/D because both studies are conducted and pursued with very similar backgrounds.

In conclusion, this prospective phase II study demonstrates that TMT for MPM meet the primary endpoints but its risk:benefit ratio is not satisfactory.

## Electronic supplementary material

Below is the link to the electronic supplementary material.

Supplementary material 1 (DOCX 13 kb)
