# Supplementary material for: Trimodality strategy for treating malignant pleural mesothelioma: results of a feasibility study of induction pemetrexed plus cisplatin followed by extrapleural pneumonectomy and postoperative hemithoracic radiation (Japan Mesothelioma Interest Group 0601 Trial)
Source: Int J Clin Oncol. 2015 Nov 17;21:523–30. doi: 10.1007/s10147-015-0925-1 (PMC4901093; doi:10.1007/s10147-015-0925-1)
Supplement: Supplementary file 1 — Supplementary material 1 (DOCX 13 kb) [file 10147_2015_925_MOESM1_ESM.docx]

Table 6 Comparison of prospective multicenter phase II trials of TMT with induction Pem plus Cis followed by EPP and RT

_________________________________________________________________________________

US EORTC 08031 JMIG 0601

Krug, et al^19^ Van Schil, et al^20^ present study

Patients/Institutions 77/9 59/11 42/17

Clinical stage T1-3N0-2M0 T1-3N0-1M0 T1-3N0-2M0

Histology any any any

Induction chemotherapy Pem 500mg/m2 Pem 500mg/m2 Pem 500mg/m2

+ Cis 75 mg/m2 + Cis 75 mg/m2 + Cis 60 mg/m2

4 courses 3 courses 3 courses

Postoperative RT h-RT (54 Gy) or IMRT h-RT (54 Gy) h-RT (54 Gy)

Induction chemotherapy

Started 77 57 42

Completed 64 (83% of 77) 55 (93% of 59) 39 (93% of 42)

Response rate 33% 44% 33%

EPP

Started 57 46 33

Completed 54 (70% of 77) 42 (71% of 59) 30 (71% of 42)

MCR not reported 40 (68% of 59) 30 (71% of 42)

Postoperative RT

Started 44 (57% of 77) 38 (64% of 59) 19 (45% of 42)

Completed 40 (52% of 77) 37 (63% of 59) 17 (40% of 42)

TMT completion rate 51.9% (40/77) 62.7% (37/59) 40.5% (17/42)

Surgical mortality rate (30-day) 3.7% (2/54) 6.5% (3/42) 3.3% (1/30)

Treatment-related mortality 3.9% (3/77) 11.9% (7/59) 9.5% (4/42)

OS months

ITT 16.8 (13.6-23.2) 18.4 (15.6-32.9) 19.9 (14.2-27.3)

TMT completed 29.1 33.0 39.4

PFS months 10.1 (8.6-15.0) 13.9 (10.9-17.2) 11.0 (2.2-31.5)

_________________________________________________________________________________

Pem: pemetrexed; Cis: cisplatin; h-RT: hemithoracic radiation therapy; EPP: extrapleural pneumonectomy; TMT: trimodality therapy
